# Prevalence of depressive symptoms among teachers in the Tshwane Metropolitan Municipality, South Africa

**DOI:** 10.4102/sajpsychiatry.v32i0.2532

**Published:** 2026-02-25

**Authors:** Kebogile E. Mokwena, Khomotso C. Maaga, Moreoagae B. Randa

**Affiliations:** 1 Department of Public Health, School of Healthcare Sciences, Sefako Makgatho Health Sciences University, Pretoria, South Africa

**Keywords:** teachers, mental disorders, depressive symptoms, workplace, South Africa

## Abstract

**Background:**

Depression is a significant contributor to the overall burden of mental disorders. The workplace has been identified as a notable source of depression, which includes the school environment. Mental disorders among teachers, especially if undiagnosed and untreated, contribute to compromised teaching and learning outcomes.

**Aim:**

The purpose of this study was to determine the prevalence of depressive symptoms among teachers in the Tshwane Municipality, South Africa.

**Setting:**

Tshwane Municipality, South Africa.

**Methods:**

The Patient Health Questionnaire was used to screen depressive symptoms; a quantitative questionnaire was used to collect socio-demographic data; and Stata 14 was used to analyse the data.

**Results:**

Most of the participants (*n* = 299, 75.13%) were female and married (*n* = 223, 56.03%), with a mean age of 39.5 years (standard deviation [SD] 12.4). The prevalence of depressive symptoms was 50.25% (*n* = 200). Being married, teaching Grade 12 and not seeking professional mental help within the previous 6 months were associated with the presence of depressive symptoms (*p* ≤ 0.05).

**Conclusion:**

The findings of this study indicate a high prevalence of depressive symptoms among teachers in the Tshwane Municipality, highlighting depression as a significant mental health concern within the school environment.

**Contribution:**

This study confirmed the high prevalence of depressive symptoms among both primary and high school teachers and across the three districts of the Tshwane Municipality.

## Introduction

The increasing prevalence of mental disorders impacts negatively on human health and societal welfare.^1^ Globally, mental disorders rank among the top 10 leading causes of morbidity, and predictions from the World Health Organization indicate that mental health disorders will become the foremost cause of disability globally by 2030.^2,3^

Depressive and anxiety disorders are the two most common mental disorders, which contribute significantly to the burden of mental illness. Together, they directly affect an estimated half a billion people, with a notable over-representation among women.^4^ Various factors play a role in influencing the onset of depression. These factors encompass anxiety, early adversity,^5^ as well as socio-economic status and demographic characteristics.^6^

Compared to other African nations, South Africa exhibits a higher prevalence of mental disorders,^7,8^ which makes mental health a focus area in South African health services, although it has not received the necessary prioritisation.^8^ Addressing mental health challenges requires enhancing policies related to the detection, management and monitoring of mental disorders. The availability of easy-to-use mental health screening tools enables the integration of mental health assessment in primary health care services.^9,10^ Routine screening for mental disorders could significantly contribute to detection and consequent treatment and other supporting interventions.^11^

The workplace has been identified as a source of depression among many people,^11^ which includes the school environment as a workplace for teachers.^12,13^ Although the teaching profession can be extremely rewarding, it is also recognised as highly stressful and demanding.^12^ Teachers encounter a range of work-related pressures, such as work overload, which occurs when teachers are assigned more tasks than they can realistically manage within the available time and resources, as well as teachers being expected to manage multiple demands or teach large or many classes. Emotional labour occurs when teachers are required to display cheerfulness or compassion regardless of their true emotions. Emotional labour contributes to a lack of psychological safety, which means that the teachers lack the safety of freely discussing ideas or asking questions without fear of adverse consequences, which raises concerns about the mental health of teachers in both elementary and secondary schools.^14^ A recent South African study identified unfavourable conditions and risk factors associated with depression among teachers to be mostly workplace-related, such as the socio-economic setting of the school, the learner-to-teacher ratio and the subjects taught by the teacher.^15^ Associated personal factors such as gender, marital status and race have been reported.^15,16^

The many challenges in the education system in South Africa include the high rate of teacher absenteeism,^17,18^ which contributes to poor teaching and learning outcomes.^18^ Absenteeism exists across the education sector regardless of teaching experience, workplace conditions or student population,^19^ which suggests that absenteeism is related to the teaching profession itself or related conditions.

Absenteeism has an inverse relationship with job satisfaction,^19,20^ which implies that high absenteeism among teachers may be an indication of experienced poor working conditions. On the other hand, absenteeism among teachers has been identified as a manifestation of various mental health challenges which may not have been identified yet.^21^ Other studies have reported mental health challenges as a significant contributing factor to teacher absenteeism.^22,23^ This suggests that problematic absenteeism among teachers can be reduced by attending to the mental health of teachers, with resultant improvement in health and resultant positive changes to teaching and learning outcomes. Moreover, the absenteeism of some teachers increases pressure on the remaining teachers, as they take on extra responsibilities to attend to all learners.^23^ This leads to burnout and depression, and thus mental pressure on others, and more cases of absenteeism.^24^

Presenteeism, which is the phenomenon of reporting for duty despite being unwell, also compromises the quality of teaching and interrupts teacher–student relationships, which further worsens presenteeism among teachers.^25^ There is an association between the quality of the teacher–student relationship and the well-being of students,^26^ which places the well-being and functionality of teachers at a level worthy of attention for the influence on the well-being of the children they interact with. Poor mental health of teachers is therefore much more consequential, and thus a priority in the considerations for teaching and learning outcomes. The purpose of this study was to screen for depression among teachers in three school districts of the Tshwane Municipality, South Africa.

## Research methods and design

### Study design and settings

The study used a cross-sectional and quantitative survey design. The population of the study consisted of teachers employed in primary and high schools in the three education districts in the Tshwane Metropolitan Municipality, these being Tshwane South, North and West.

### Sample

All three school districts in the Tshwane Metropolitan Municipality were included in the study. Stratified sampling was used at the district level, and the hat method was used to randomly select 15 schools from each district for participation. However, because of insufficient participation, an additional five schools were selected to meet the required sample size. Within each identified school, all education professionals, which included teachers, principals and deputy principals, who had been practising teaching for a minimum of 1 year, were invited to participate in the study.

Using the Raosoft sample size calculator,^27^ for the estimated 21 000 teachers in the Tshwane Metropolitan Municipality, a 5% margin of error, a 95% confidence interval and a 50% response distribution, a minimum sample size of 379 was calculated.

### Data collection

The Patient Health Questionnaire (PHQ-9), initially developed by Kroenker, Spitzer and Williams,^28^ was used to collect data from the sample. The tool has been globally confirmed for both validity and reliability.^29^ It has been recommended for use in resource-constrained settings,^30^ including several African countries^31,32^ and South Africa.^15,32,33^ It has high sensitivity and specificity,^33,34^ displays stability at repeated measurements and can be used to monitor changes of symptoms over time. The PHQ-9 tool asks participants about depression-related symptoms over the previous 2 weeks, with options ranging from ‘0’ (not at all) to ‘3’ (nearly every day). The questions asked related to the participant’s sleeping and eating patterns, ability to concentrate on tasks, and thoughts related to self-harm. Scores from the nine questions give a maximum score is 27. A questionnaire was used to collect socio-demographic data. The questionnaire asked about socio-demographic information (i.e. age, gender and marital status) of the participants as well as work-related demographics (i.e. conditions of employment and learner–teacher ratio).

Data collection occurred at the school premises, and participants who agreed to participate were assembled in an office, were given an explanation about the study and were allowed to ask questions or seek clarifications regarding the study. When ready, they were requested to provide informed consent by signing the informed consent form, which was followed by the administration of the socio-demographic questionnaire and the PHQ-9 scale.

### Statistical analysis

The raw data were captured into Microsoft Excel, cleaned, coded, and exported to Stata version 14 (Stata Corporation, College Station, Texas, United States) for analysis. Demographic data were analysed descriptively and expressed as means, medians and standard deviations (SD).

To determine the prevalence of depressive symptoms, PHQ-9 scores obtained were used to classify the participants as not depressed for scores of 0 to 4 or depressed for scores of 5 and above. The severity of depressive symptoms was further categorised as mild for scores of 5 to 9, moderate for scores of 10 to 14, moderately depressed for scores of 15 to 19, and severely depressed for scores above 19. The Pearson Chi-squared test was used to explore an association between the scores of the PHQ-9 and various socio-demographic variables (*p* ≤ 0.05). To meet statistical testing assumptions, all numeric data were coded as categorical data (e.g. depression scores were coded as depressed or not depressed). Multivariate analysis was conducted using a binary logistic regression model for variables which were significantly associated with depression (*p* ≤ 0.05). Categorical data that were significant during Pearson Chi-squared tests were recoded as numeric to meet statistical testing assumptions for logistic regression.

### Ethical considerations

Ethical clearance to conduct this study was obtained from the Research Ethics Committee of Sefako Makgatho Health Sciences University (Ref. No. SMUREC/H/62/2019:IR). The Gauteng provincial office of education and the management of each of the three districts provided written permission for the study to be conducted. The management of each school permitted the study to be conducted, and all participants provided individual written informed consent.

In accordance with ethical research practices, participants who experienced psychological distress during the study were referred to the South African Depression and Anxiety Group (SADAG) for professional support. Data confidentiality was ensured by keeping the data in a password-protected electronic file on the desktop of the principal investigator. All data collectors signed a confidentiality document regarding the data.

## Results

### Profile of the schools

A total of 398 participants from Tshwane South (*n* = 207, 52.01%), North (*n* = 122, 30.65%) and West (*n* = 69, 17.34%) from 20 schools, consisting of 7 (42.23%) primary and 13 (57.77%) high schools, participated in the study. The number of participants in each school ranged from 8 to 120, with an average of 27. The quintile rankings of the schools ranged from 1 to 5, with 23% employed at quintiles 1 to 3 and 77% employed at quintiles 4 and 5.

### Socio-demographic characteristics

The largest proportion of the participants were female(*n* = 299, 75.13%) and married (*n* = 223, 56.03%). Their ages ranged from 20 years to 68 years, with a mean of 39.5 years and SD of 12.4. Only a few of the participants (*n* = 19, 7.92%) had sought professional help for mental health within the previous 6 months. The rest of the socio-demographic variables are shown in Table 1.

**TABLE 1:** Socio-demographic characteristics (*N* = 398).

Factors	Frequency	%
**Age (Years)**		
20–40	201	50.50
41–68	163	40.95
Unknown	34	8.54
**Gender**		
Female	299	75.13
Male	99	24.87
**Home language**		
Afrikaans	179	44.97
Setswana	62	15.58
Sepedi	40	10.05
English	38	9.55
Isizulu	29	7.29
Xitshoga	12	3.02
IsiXhosa	11	2.76
IsiSwati	7	1.76
Tshivhenda	1	0.25
IsiNdebele	6	1.51
Sesotho	4	1.01
Other	2	0.50
**Marital status**		
Married	223	56.03
Single	147	36.93
Divorced	16	4.02
Widowed	12	3.02
**Sought mental health professional help in past 6 months (*n* = 240)**		
No	221	92.08
Yes	19	7.92

Source: Adapted from Mokwena K, Mokgatle M. Occupation-related anxiety symptoms among teachers in Tshwane Metropolitan Municipality, South Africa. Perspect Educ. 2025;43(1):157–171. https://doi.org/10.38140/pie.v43i1.7491.^35^

Note: Mean 39.5; SD 12.4; Min 20; Max 68.

### Variables related to the workplace

Over half of the sample had a bachelor’s degree (*n* = 238, 59.80) as their highest level of education, and had a permanent contract basis (*n* = 326, 82.95) with an average teaching experience of 13.40 years. Table 2 below shows the rest of socio-demographic variables that are related to the workplace.

**TABLE 2:** Factors related to the workplace.

Factors	Frequency	%
**Highest qualifications obtained (*n* = 398)**		
Bachelor’s degree	238	59.80
Postgraduate diploma	68	17.09
Diploma	66	16.58
Master’s degree	14	3.52
Doctoral degree	2	0.50
Missing	10	2.51
**Conditions of appointment (*n* = 393)**		
Permanent	326	82.95
Temporary	67	17.05
**Level of appointment (*n* = 385)**		
Teachers	349	88.35
Heads of department	34	8.61
Deputy principals	7	1.77
Principals	5	1.27
**Years in teaching (*n* = 398)**		
≤ 14	234	58.79
˃ 14	164	41.21
**Years in the particular school (*n* = 398)**		
≥ 8	239	60.05
˃ 8	159	39.95
**Number of learners per teacher (*n* = 398)**		
≤ 25	210	52.76
≥ 25	188	47.24
**Number of subjects responsible for (*n* = 366)**		
1–2	243	66.40
≥ 3	123	33.60
**Subjects taught**		
Languages	162	40.71
Maths	113	28.39
Commerce	31	7.79
Arts	32	8.04
Life Sciences	80	20.10
Natural Sciences	19	4.77
Social Sciences	18	4.52
Other	83	20.85
**Number of grades responsible for (*n* = 294)**		
1–2	175	59.52
≥ 3	119	40.48

Source: Adapted from Mokwena K, Mokgatle M. Occupation-related anxiety symptoms among teachers in Tshwane Metropolitan Municipality, South Africa. Perspect Educ. 2025;43(1):157–171. https://doi.org/10.38140/pie.v43i1.7491.^35^

Note: Mean 13.40; SD 10.72; Min 1; Max 45.

### Prevalence of depressive symptoms

The prevalence of depressive symptoms was 50.25% (*n* = 200), with the majority (*n* = 105, 26.38%) exhibiting mild compared to a few exhibiting severe symptoms (*n* = 10, 2.51%) Table 3 below shows the prevalence andcategories of depressive symptoms.

**TABLE 3:** Categories of depressive symptoms.

Depression category	Frequency	%
Not depressed	198	49.75
Mild	105	26.38
Moderate	61	15.33
Moderately severe	24	6.03
Severe	10	2.51

**Total**	**398**	**100**

The Pearson Chi-squared test of associations was used to explore which socio-demographic and workplace-related factors were significantly associated with symptoms of depression, and it was found that marital status, grade taught as well as whether the participant had sought professional mental help within the previous 6 months were associated (*p* ≤ 0.05), as displayed in Table 4.

**TABLE 4:** Factors associated with depressive symptoms.

Factors	Frequency	%	Depressed	%	Not depressed	%	Chi^2^	*P*-value
**Marital status**							8.99	0.03
Divorced	16	4.02	6	3.00	10	5.05	-	-
Married	223	56.03	105	52.50	118	59.60	-	-
Single	147	36.93	86	43.00	61	30.81	-	-
Widowed	12	3.02	3	1.50	9	4.55	-	-
**Sought mental health-related professional help in the past 6 months**							8.64	0.00
No	221	92.08	97	86.61	124	96.88	-	-
Yes	19	7.92	15	13.39	4	3.13	-	-
**Grade responsible for: Grade 11**							7.86	0.01
No	282	70.85	129	64.50	153	77.27	-	-
Yes	116	29.15	71	35.50	45	22.73	-	-
**Grade responsible for: Grade 12**							5.92	0.02
No	305	76.63	143	71.50	162	81.82	-	-
Yes	93	23.37	57	28.50	36	18.18	-	-

### Multivariate analysis

The factors that were significant at a univariate level were utilised to build a multivariate model using binary logistic regression, and all factors remained significantly associated except one, that is, participants teaching Grade 11 (Table 5). The analysis showed that participants who were married were 2.5 times more likely to exhibit elevated symptoms of depression. Additionally, those who taught Grade 12 were also 2.1 times more likely to report symptoms of depression. Finally, those who had not sought mental health-related professional help in the past 6 months were at a 3.7 times greater risk of depressive symptoms.

**TABLE 5:** Multivariate analysis.

Variable	OR	SE	*P*-value	95% CI
Marital status	2.46	0.60	0.00	1.52–3.99
Sought mental health-related professional help in past 6 months	3.70	2.23	0.03	1.13–12.08
Teaching Grade 11 classes	1.68	0.56	0.12	0.87–3.25
Teaching Grade 12 classes	2.01	0.73	0.05	0.99–4.078

OR, odds ratio; SE, standard error; CI, confidence interval.

## Discussion

This study aimed to determine the prevalence of depressive symptoms among teachers in the Tshwane Municipality, South Africa. Participants were drawn from Tshwane South, North, and West, with the majority residing in Tshwane South. The sample was predominantly female and married, with a mean age of approximately 40 years. The finding that most teachers in the sample are female confirms findings from other studies, which profile the teaching profession as being dominated by females.^15,29^ This trend has been explained by the Theory of Gender and Power, which divides women and men into gender-specific occupations,^36^ and thus renders the teaching profession to be associated with caring and femininity.^37^

The finding that female teachers have a higher prevalence than their male counterparts is aligned with established findings of higher depression prevalence among females in the general population.^38^ Although some studies have reported marriage to be a protective factor against poor mental health outcomes,^37,38^ it was interesting to find that marital status in the current study was significantly associated with elevated symptoms of depression among married teachers. The difference may be explained by the quality of the marriage, as poor marital quality, marital conflict, lack of spousal support and domestic violence may contribute towards deteriorating mental health outcomes, which may serve as a plausible explanation for the current study findings.^39^

The prevalence of depressive symptoms was just above half, which is high but similar to other studies which reported a high global prevalence of depressive symptoms among teachers. The current study rate is approximately 25% higher than national rates among the general South African population,^40^ which highlights the difficulties faced by teachers. African countries which have reported high prevalence of depression among teachers include Namibia^41^, Nigeria^42^, Ghana^43^, Tanzania^44^ and South Africa^15^. The high prevalence cuts across the education profession, from early learning practitioners^16^ to academics in higher education institutions.^45^

This study found work-related factors significantly associated with depressive symptoms at both univariate and multivariate levels, which were reported by other studies, which reported work-related factors like difficult relationships, including teachers being bullied by learners,^46,47^ as well as dealing with bullying among learners,^48^ which is rife in South African schools.^49^ Moreover, often teachers do not know how to deal with these problems which they are expected to resolve,^50,51^ which worsens the mental health of such teachers. Other workplace-related risk factors for depressive symptoms among teachers reported in other studies were the low socio-economic status of the school, the lack of a conducive school environment and the unfavourable learner-to-teacher ratio,^15^ which are a reality for many teachers in South Africa.

This study found that being responsible for teaching Grade 12 was significantly associated with symptoms of depression, which confirms the importance that the education system and South Africans place on academic performance in Grade 12. This grade is not only the last year of the schooling system, but student performance in this grade is also a measure of the academic performance of a school and a critical gateway to higher education and employment prospects.^51^ The impact of the pressure on learners is also reflected by the symptoms of depression in teachers, which suggests consideration for support for teachers responsible for teaching this grade.

The study found that seeking professional help regarding mental health is a protective factor against depressive symptoms, while failure to seek help is a risk factor. Among teachers, barriers to seeking help for mental health challenges include a lack of training in assessment of mental health as well as a lack of knowledge, skills and confidence in the area of mental health.^52,53^ The finding also suggests limited knowledge regarding where to seek help pertaining to their mental health.

Because mental health challenges are reported to be rife among teachers, employee assistance programmes should include training in both their pre- and in-service.^52^ Common undesirable behaviours relating to experiences of poor mental health include masking or ignoring the symptoms,^53,54^ which results in a worsening of the symptoms, while the productivity deteriorates.^54^ When the person can no longer mask the pain, he or she breaks down, and recovery takes a longer time. Absenteeism and presenteeism have also been linked to underlying mental health challenges, including depression, and can negatively affect teaching quality and teacher–learner relationships.^20–22,24,25^ While these outcomes were not directly assessed in this study, they represent potential occupational consequences of depressive symptoms among teachers, with implications for the broader school environment.

Teaching is one of the careers that requires optimum mental health, because the profession itself focuses on the mental development of children. The mental health of teachers therefore needs intentional attention to promote productivity in their profession. The scourge of mental health challenges among children has resulted in calls for non-mental health professionals who are in contact with children, such as teachers, to be trained to identify and mitigate such challenges among children.^56^ However, teachers cannot play a meaningful role if they themselves are struggling with mental health challenges that they are not ready, willing and able to mitigate.

### Strengths and limitations of the study

A limitation of the study is the cross-sectional design, which cannot establish causality and only establishes disease prevalence at one point in time. Additionally, the PHQ-9 scale used in the study only establishes subthreshold symptoms of depression, indicating probable depression, but is not a measure of major depressive disorder, requiring caution when interpreting the results. Moreover, the role played by volunteer bias is acknowledged, as it may have affected the results with greater participation among participants experiencing elevated symptoms of depression.

The strengths of the study include a large sample size and collecting data from various sources across 3 districts, both primary and secondary schools and from 20 schools, which enhances variability.

## Conclusion

This study confirmed the high prevalence of depressive symptoms among both primary and high school teachers and across the three districts of the Tshwane Municipality. Mental illness among teachers is costly for the teachers themselves, as well as the children who are exposed to being taught by teachers who are not in optimum health. This study and others call for the promotion of mental health in the teaching profession, with the development of employee assistance programmes with significant attention to mental health promotion.^55^ Moreover, the PHQ-9 scale used in the current study is merely a screening tool indicating probable depression; thus, future research should consider using diagnostic interviews to adequately quantify the prevalence of major depressive disorder.
